# Benefits of Probiotic Yogurt Consumption on Maternal Health and Pregnancy Outcomes: A Systematic Review

**DOI:** 10.7759/cureus.9408

**Published:** 2020-07-26

**Authors:** Andy He, Justin Chin, Christine M Lomiguen

**Affiliations:** 1 Department of Primary Care, Touro University Nevada, Henderson, USA; 2 Family Medicine, LifeLong Medical Care, Richmond, USA; 3 Medical Education, Lake Erie College of Osteopathic Medicine, Erie, USA; 4 Pathology, Lake Erie College of Osteopathic Medicine, New York, USA

**Keywords:** probiotics, pregnancy, yogurt, maternal health, probiotic yogurt, probiotic

## Abstract

The purported benefits of probiotics have been touted as adjunctive or alternative treatment to a variety of diseases. Limited studies have investigated the role of probiotic yogurt in the prevention and management of pregnancy-related adverse events. This literature review aims to analyze the benefits of probiotic yogurt on improving maternal health and pregnancy outcomes and to further identify possible areas of study. A detailed search was conducted utilizing the National Library of Medicine's MEDLINE/PubMed database. The following search terms were queried: ("probiotic" OR "probiotics") AND ("yogurt" OR "yoghurt") AND ("pregnancy"). All articles identified by this search strategy were retrieved in their entirety, analyzed for relevance, and thoroughly reviewed for additional studies. All data were accessed in March 2020. The review process revealed 13 manuscripts that met inclusion criteria for review, the majority (n=10) of which were clinical trial reports. The manuscripts were further classified and grouped broadly by study outcomes. The consumption of probiotic yogurt was found to improve metabolic, inflammatory, and infectious outcomes of pregnancy. Studies on the consumption of probiotic yogurt appear to have many positive benefits, ranging from improving metabolism to decreasing preterm births. While its mechanism is still largely unclear, probiotic yogurt holds promise as a nutritional, global pregnancy supplement. Future research should be conducted and may consider detailed study of more fermented foods that offer categorization as a probiotic. Additional funding and research conducted in other countries may also clarify the effects of probiotic yogurt consumption on pregnancy outcomes.

## Introduction and background

Probiotics are defined as live microorganisms that are consumed with the goal of promoting health through improvement or restoration of gastrointestinal flora [1]. Typically consisting of an ever-increasing list of bacteria and yeasts, most notably Lactobacillus, Bifidobacterium, and Saccharomyces, purported benefits have been touted as adjunctive or alternative treatment to a variety of diseases [1,2]. However, due to potential teratogenic effects of common prescription, over-the-counter, and supplementary medications on organogenesis and embryonic development, the use of probiotics during pregnancy has become a topic of increasing interest for peripartum healthcare professionals and new mothers alike [3,4]. Pregnancy is a period of high nutritional demand, hormonal fluctuations, and emotional lability, to which probiotics have been linked to providing various benefits in both anecdotal experiences and validated research [5,6]. While the most common strains found in probiotics have not been found to cause miscarriage or cross the placental barrier, increased attention given to probiotics demands an in-depth literature review. 

In the year 2000, the United Nations Millennium Development Goals committed leaders of the world to eight goals, the fifth of which aimed to improve maternal health [7]. In light of the rise of social media and the dramatic spread of information facilitated by the internet, physicians are faced with more inquiries as families explore the use of probiotics in various pregnancy-related symptoms and complications [8-10]. Yogurt has been commonly cited as a prospective option for further trials due to its worldwide availability, nutritional benefit, and potential for live microorganism delivery [11]. Limited studies have investigated the role of probiotic yogurt in the prevention and management of adverse pregnancy-related events such as gestational diabetes, preeclampsia, congenital anomalies, preterm birth, and infantile metabolic disorders [5,12,13]. This literature review aims to analyze the benefits of probiotic yogurt on improving maternal health and pregnancy outcomes and to further identify possible areas of study. 

Methods

A detailed search was conducted utilizing the National Library of Medicine's MEDLINE/PubMed database, with the objective to identify all articles published in the English language between January 1980 and February 2020. The following search terms were queried: ("probiotic" OR "probiotics") AND ("yogurt" OR "yoghurt") AND ("pregnancy") in which combinations yielded 18 articles with their respective abstracts. The initial review focused on publications within the last decade, however, older manuscripts were not excluded. All articles identified by this search strategy were retrieved in their entirety, analyzed for relevance, and thoroughly reviewed for additional studies not found by the previous method. All data was accessed in March 2020, with 18 articles meeting initial inquiry. Further review revealed five articles that were duplicated, not in English, or not relevant to probiotic pregnancy outcomes; these were subsequently excluded. Of the 13, 12 studies and one literature review were included in which study type, location, number of participants, trimester of pregnancy, and research conclusions were compared and analyzed for overarching trends and messages. 

## Review

Characteristics of studies

Qualitative data for the systematic review of the 13 articles in regard to the consumption of probiotic yogurt on maternal health and pregnancy outcomes can be found in Table 1. In regard to the types of studies, the majority of studies performed were clinical trials (n=10) with two case-control studies and one literature review. Among the clinical trials, seven considered themselves to be randomized controlled clinical trials with two studies self-labeling as open-label clinical trials. The remaining clinical trial was identified as being an observational clinical trial. Two studies were identified as case-control studies. Of the 12 case-control studies and clinical trials, 11 had sample sizes over 30 in each of the experimental and control groups. All studies evaluated a period of probiotic yogurt consumption during pregnancy. One study also included probiotic yogurt consumption prior to pregnancy. Six of the studies solely focused on the third trimester of pregnancy.

**Table 1:** Summary of probiotic yogurt studies in pregnancy Assessment of type of study performed, location, number of participants, and period of pregnancy in which the study was performed. Data is further grouped into types of study.

Author and Year of Article	Type of Study	Location of Study	Participants (experimental/control)	Period of Pregnancy
Case Control Studies (2)				
Chen, et al., 2019 [5]	Case-control	China	(123/126)	Pre-pregnancy and Pregnancy
Celik, et al., 2019 [12]	Case-control	Turkey	(84/56)	Pregnancy
Clinical Trials (10)				
Kriss, et al., 2018 [14]	Clinical trial - observational	Mexico	(965)	Mid-pregnancy
Mirghafourvand, et al., 2016 [6]	Randomized controlled clinical trial	Iran	(30/30)	Pregnancy
Bisanz, et al., 2015 [15]	Open-label clinical trial	Tanzania	(26/30)	Pregnancy
Bisanz, et al., 2014 [11]	Open-label clinical trial	Tanzania	(60)	2^nd^ and 3^rd^ trimester
Asemi, et al., 2013 [16]	Randomized controlled clinical trial	Iran	(70)	3^rd^ trimester
Asemi and Esmaillzadeh, 2013 [17]	Randomized controlled clinical trial	Iran	(70)	3^rd^ trimester
Asemi, et al., 2012 [18]	Randomized controlled clinical trial	Iran	(70)	3^rd^ trimester
Asemi, Jazayeri, et al., 2012 [19]	Randomized controlled clinical trial	Iran	(70)	3^rd^ trimester
Asemi, et al. 2011 [13]	Randomized controlled clinical trial	Iran	(70)	3^rd^ trimester
Hantoushzadeh, et al., 2012 [20]	Randomized controlled clinical trial	Unable to assess	(310)	3^rd^ trimester
Literature Review (1)				
Othman, et al., 2007 [21]	Review	N/A	4 randomized controlled trials, 2 included	

The articles were further classified and grouped broadly into study outcomes the authors were investigating as noted in Table 2. The “metabolic” category consists of studies (n=5) evaluating the consumption of probiotic yogurt on gestational diabetes mellitus, insulin, heavy metal toxicity, calcium, and serum lipid profiles. The “inflammatory” category (n=3) comprises studies that evaluated the outcomes of consuming probiotic yogurt on atopic dermatitis, levels of oxidative stress markers, and inflammatory cytokines. Probiotic yogurt effects on bacterial vaginosis were classified into an “infectious” group (n=2). Lastly, three articles were unable to be categorized into the previous three groups, but addressed preterm delivery, constipation, and the microbiota of various body sites.

**Table 2:** Summary of probiotic yogurt studies with the type of outcomes and conclusions Type of study outcomes and specific conclusions of studies. Categorized by the type of study outcome. Abbreviations: AST, aspartate transaminase; ALT, alanine transaminase; hs-CRP, high sensitivity C-reactive protein; and TNFa, tumor necrosis factor-alpha

Author and Year or Article	Type of Study Outcome	Conclusions
Metabolic (5)		
Chen, et al., 2019 [5]	Metabolic	Reduced risk of gestational diabetes mellitus
Bisanz, et al., 2014 [11]	Metabolic	Protective effects against increased mercury and arsenic blood levels
Asemi, et al., 2013 [16]	Metabolic	Maintained serum insulin levels and may prevent insulin resistance development
Asemi, et al., 2012 [18]	Metabolic	No effect of serum lipid profiles compared to conventional yogurt
Asemi and Esmaillzadeh, 2013 [17]	Metabolic	Maintained serum calcium levels, but no significant changes seen with iron, AST, and ALT serum levels
Inflammatory (3)		
Celik, et al., 2019 [12]	Inflammatory	Reduced risk of infantile atopic dermatitis
Asemi, Jazayeri, et al. 2012 [19]	Inflammatory	Increased levels of erythrocyte glutathione reductase, but did not affect other markers of oxidative stress
Asemi, et al., 2011 [13]	Inflammatory	Significant decrease of hs-CRP, but no effect on TNFa
Infectious (2)		
Hantoushzadeh, et al., 2012 [20]	Infectious	Good efficacy of treating bacterial vaginosis and may decrease preterm birth
Othman, et al., 2007 [21]	Infectious	Appears to treat infections during pregnancy, but lack sufficient data to evaluate impacts on preterm birth
Other (3)		
Kriss, et al., 2018 [14]	Other - Preterm Delivery	Reduced preterm delivery among non-overweight women with higher prenatal yogurt consumption
Mirghafourvand, et al., 2016 [6]	Other - Constipation	Probiotic and conventional yogurt can improve symptoms of constipation
Bisanz, et al., 2015 [15]	Other - Microbiome	No effect on mother’s microbiota at any body site, but changed microbiota of newborn feces

Review of studies

Among the 13 reviewed manuscripts, the consumption of probiotic yogurt has been found to improve metabolic, inflammatory, and infectious outcomes of pregnancy as well as affect preterm delivery, constipation, and changes in microbiota.

Probiotic yogurt was found to have largely beneficial effects on metabolism and metabolic changes, with moderation of insulin levels, prevention of insulin resistance, and decreased incidence of gestational diabetes [5,16,19]. Maintenance of serum calcium levels during the third trimester of pregnancy has been seen with probiotic yogurt consumption, which contrasts the expected decrease in levels due to fetal demand [17]. In both cases, probiotic yogurt prevented complications associated with calcium deficiency such as preeclampsia, abnormal fetal programing, rickets, hypocalcemia, and tetany [16,17]. Due to the affinity of various bacterial strains to heavy metals, probiotic yogurt prevented the uptake of arsenic and mercury in model blood samples [11]. This opens up the possibility that probiotic yogurt may be used in lieu of short-term dimercaptosuccinic acid or ethylenediaminetetraacetic acid for populations that require long-term chelation during pregnancy [11]. Due to the complexity of diet relative to cultural and ethnic practices in pregnancy, no significant changes to lipid profiles could be solely attributed to probiotic yogurt, however conventional and probiotic variants have been found to decrease low-density lipoproteins, high-density lipoproteins, and triglyceride serum concentrations in the general public [18]. 

Pregnancy is a time of high inflammation as increased oxygen demand creates elevated oxidative stress. Antioxidant and anti-inflammatory properties have been seen in maternal and infant studies status post-consumption of probiotic yogurt [19]. Decreased levels of C-reactive protein coupled with antiphlogistic markers of erythrocyte glutathione reductase, plasma glutathione, erythrocyte, glutathione peroxidase, and serum 8-oxo-7,8-dihydroguanine have been tied to probiotic consumption [13,19]. Such markers have been shown to extend beyond maternal use and are present during early infant life. Coupled with a varied diet and other fermented foods, probiotic yogurt has been shown to reduce the risk of developing atopic dermatitis in early infancy [12]. Fecal studies have demonstrated the role of probiotics such as yogurt in the promotion of beneficial gut flora variants and healthy gastrointestinal maturation [15]. The lack of standard formulations and serotypes, however, have hindered the generation of overarching conclusions as various trials have shown limited or inconclusive effects of probiotic yogurt on tumor necrosis factor-alpha and other markers of oxidative stress and inflammation [13,19].

The effects of probiotic yogurt were also evaluated in reference to the treatment of bacterial vaginosis and preventing preterm labor, as 30%-50% of preterm births are thought to be from maternal infection [20,21]. While the current mechanism of action is largely unknown, probiotics have been shown to be a viable alternative to antibiotics for bacterial vaginosis in pregnancy through the displacement of pathogens and interference of the inflammatory immune cascade [14,21]. Nonetheless, conflicting studies have also emerged in which research showing improved bowel performance is pitted against minimal changes in maternal microbiomes [6,15]. Common study limitations include cost, location, and inability to elucidate a singular mechanism to which the effects of probiotic yogurt can be ascribed. The location of these studies reviewed also comprises largely of studies in Iran (n=6) and Tanzania (n=2), which can call to question the demographic makeup and generalizability of results [13,16-19]. Additional funding and research in other countries may provide increased insight into the effects of consuming probiotic yogurt during pregnancy. As speculation continues with the mechanism by which probiotics and probiotic yogurt affect the peripartum period, larger research initiatives and collaborations are required to address the growing trend of holistic, non-pharmaceutical based care in pregnancy.

## Conclusions

Studies on the consumption of probiotic yogurt appear to have many positive benefits. These benefits extend into improving metabolism, reducing inflammation, preventing infection, and improving pregnancy outcomes by decreasing preterm births. Probiotic yogurt holds promise as a nutritional, readily available product that may benefit pregnant women globally. Future research should be conducted and may consider detailed study of more fermented foods that offer categorization as a probiotic, including kimchi, natto, pickles, olives, etc. Additional funding and research conducted in other countries may also clarify the effects of consuming probiotic yogurt on pregnancy outcomes.
